# Supplementary material for: Beta rhythmicity in human motor cortex reflects neural population coupling that modulates subsequent finger coordination stability
Source: Commun Biol. 2022 Dec 15;5:1375. doi: 10.1038/s42003-022-04326-4 (PMC9755311; doi:10.1038/s42003-022-04326-4)
Supplement: Supplementary file 2 — Supplementary Information [file 42003_2022_4326_MOESM2_ESM.pdf]

Supplementary Information

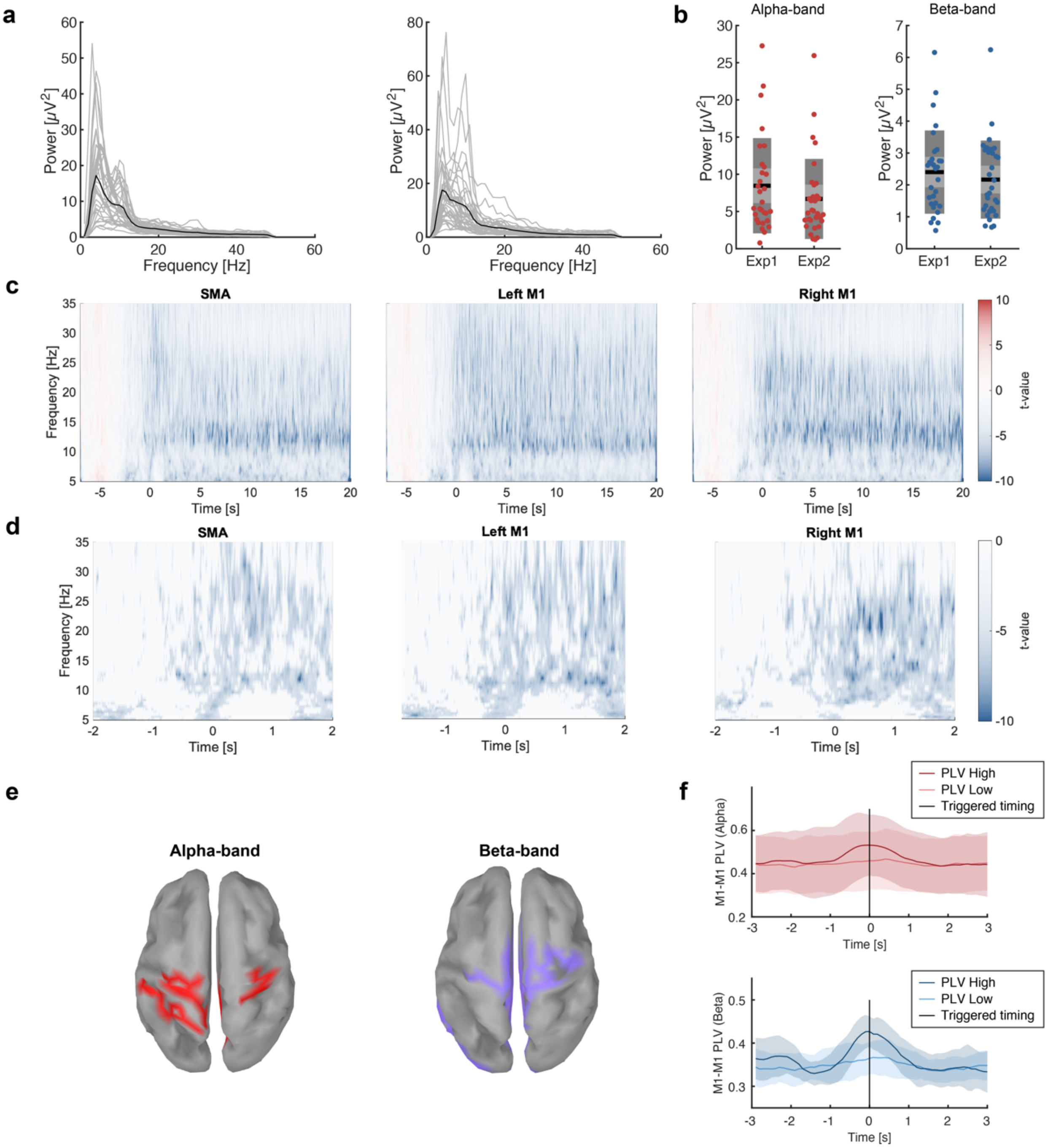

Supplementary Fig. 1 Quality assurance of EEG signals

a Power spectral density (PSD) of EEG signals over the supplementary motor area (SMA). Black and gray lines indicate the average PSD and data from each participant, respectively. *Left*: Data from participants in experiment 1. *Right*: Data from participants in experiment 2. **b** Comparison of signaling power between experiments. No significant differences were observed in overall signal strength in the frequency of interest (two-sample t-test, alpha: $t(30) = 1.20$, $p = 0.236$; beta: $t(30) = 0.74$, $p = 0.46$). **c** Time-frequency representation of source activities in the region of interest. *Left*: SMA, *Middle*: left primary motor cortex (M1), *Right*: right M1. Time points at which ERSP magnitude exhibited statistically significant differences from 0 (t-test $p < 0.05$, Bonferroni corrected) are colored. **d** Pre-movement ERSP responses at SMA (Left), left M1 (Middle), and right M1 (Right). **e** Cortical surface representation of task-related responses. *Left*: alpha-band. *Right:* beta-band. Vertices that demonstrated statistically significant differences from 0 (*t*-test, $p < 0.05$, Bonferroni corrected) are colored. **f** Time courses of online calculated hemispheric phase-locking values in alpha (Top) and beta-band (Bottom). The triggered timing was determined when PLV exceeded the threshold determined at the beginning of each session. Since the online PLV-triggered algorithm only uses the ready period data, the trend of PLV was not considered and a negative peak in PLV low condition was not found due to the cancellation of rising and falling flanks.

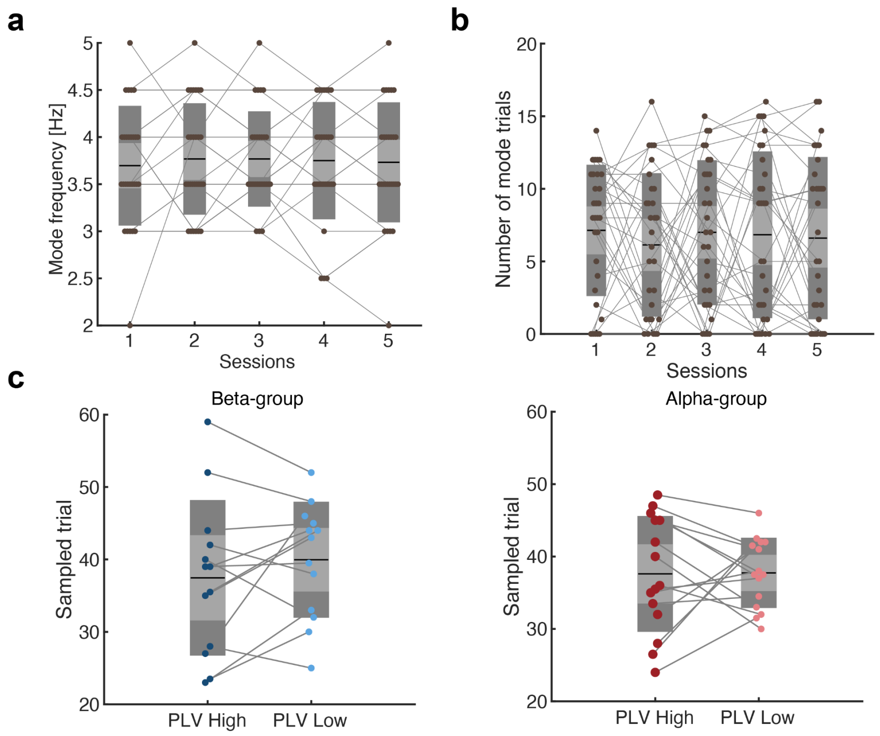

Supplementary Fig. 2 Changes in behavioral performance in experiment 1

a Changes in mode frequency among sessions. The mode frequency within a session was compared using one-way ANOVA ($F(4,135) = 0.07$, $p = 0.99$), suggesting that the overall performance was consistent during state-dependent sessions. **b** Changes in the number of mode trials, which presented an instructive sound at mode frequency of trials in all sessions. One-way ANOVA did not exhibit significant difference $F(4,145) = 0.23$, $p = 0.95$. **c** Comparison of trial numbers from which data of each condition were sampled. *Left*: comparison in the beta-band group paired *t*-test, $t(12) = -1.15$, $p = 0.273$), *Right*: comparison in the alpha-band group (paired *t*-test, $t(14) = -0.06$, $p = 0.95$).

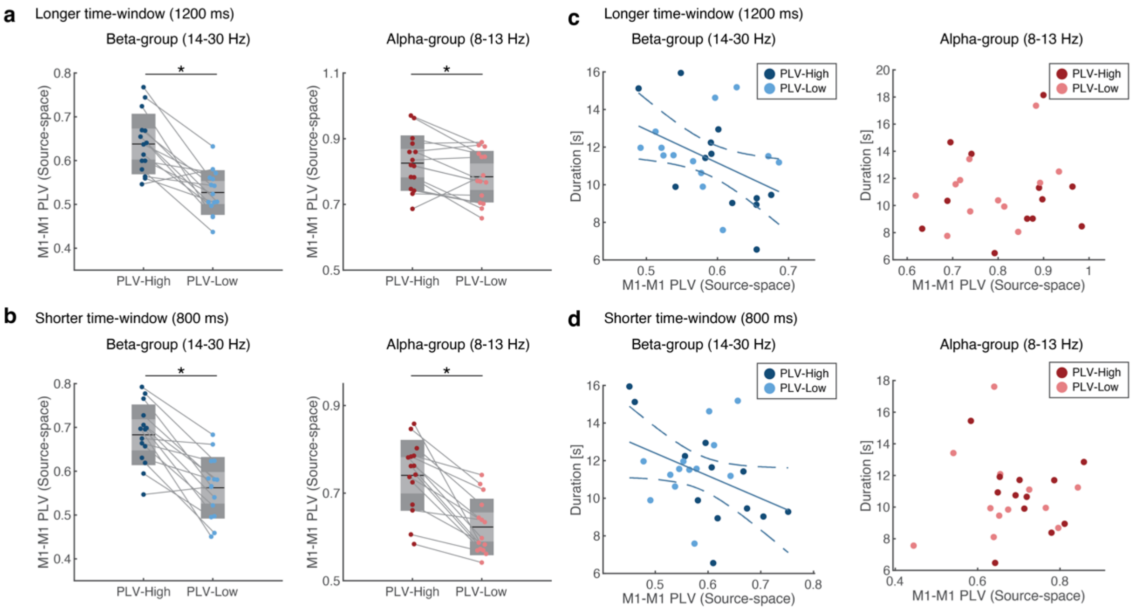

Supplementary Fig. 3 Supplementary analysis using alternative time-windows in Experiment 1

a Comparison of phase-locking values (PLVs) of bilateral primary motor cortices (M1) between two conditions, calculated with a longer time-window (1200 ms). Groups based on M1-M1 PLVs in both beta- and alpha-bands exhibited significant differences in high and low conditions (paired t-test, *: p < 0.05, beta-group: t(14) = 5.70, d = 1.47, CI_{95} = [0.7 2.20]; alpha-group: t(14) = 2.63, p = 0.02, d = 0.68, CI_{95} = [0.1 1.23]). **b** Comparison of M1-M1 PLVs between two conditions, calculated with a shorter time-window (800 ms). Groups based on M1-M1 PLVs in both beta- and alpha-bands exhibited significant differences in high and low conditions (paired t-test, *: p < 0.05, beta-group: t(14) = 6.36, d = 1.63, CI_{95} = [0.84 2.42]; alpha-group: t(14) = 6.28, d = 1.62, CI_{95} = [0.83 2.39]). **c** Correlation analysis between M1-M1 PLV (longer-time window) and duration (repeated measures correlation test, beta: r = −0.57, p = 0.04; alpha: r = −0.15, p = 0.62). Solid and dotted lines indicate linear regression and a 95% confidence interval, respectively. **d** Correlation analysis between M1-M1 PLV (shorter-time window) and duration (repeated measures correlation test, beta: r = −0.59, p = 0.03; alpha: r = −0.27, p = 0.37). Solid and dotted lines indicate linear regression and a 95% confidence interval, respectively.

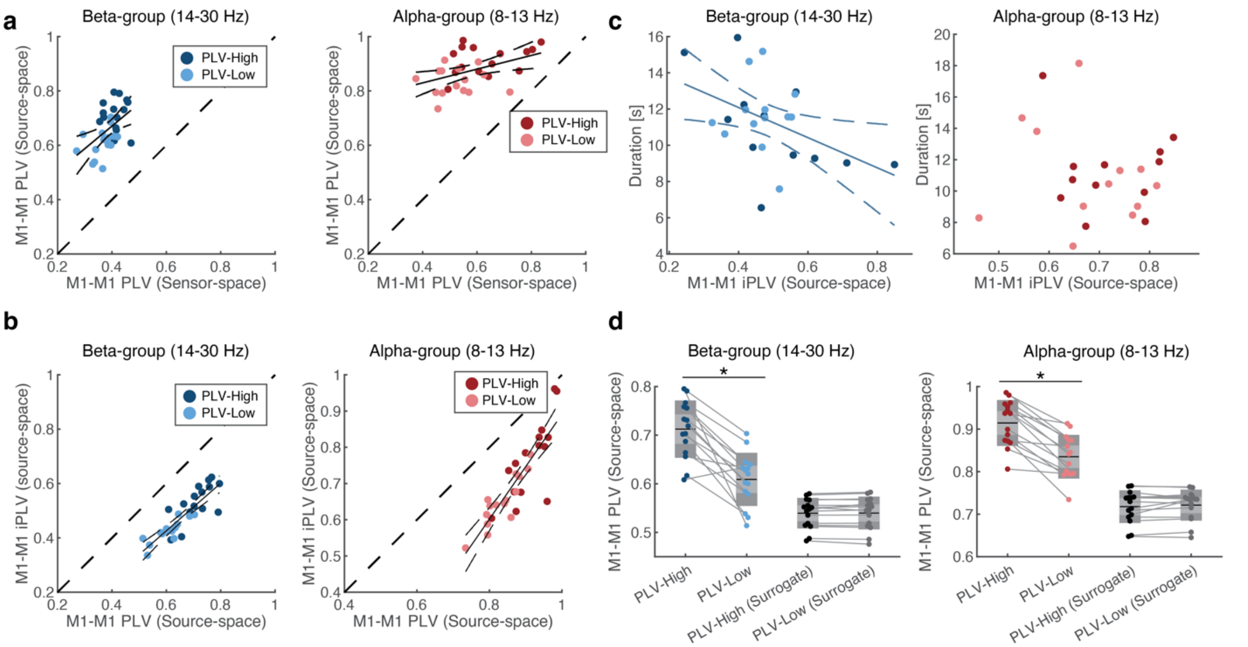

Supplementary Fig. 4 Supplementary analysis using surrogate data and alternative metric of phase synchrony

a Comparison of M1-M1 PLV derived from sensor- and source-space signals. Pearson's correlation test was employed to test the consistency between the two datasets (Beta-group, r = 0.50, p = 0.004, CI_{95} = [0.17, 0.72], Alpha-group, r = 0.45, p = 0.013, CI_{95} = [0.10, 0.70]). Solid and dotted lines indicate linear regression and a 95% confidence interval, respectively. **b** Comparison of M1-M1 PLV and imaginary-PLV (iPLV) derived from source-space signals. Pearson's correlation test was employed to test the consistency between the two datasets (Beta-group, r = 0.86, p<0.001, CI_{95} = [0.72, 0.93]; Alpha-group, r = 0.86, p < 0.001, CI_{95} = [0.73 0.93]). **c** Correlation analysis between M1-M1 iPLV and duration of anti-phase tapping (repeated measures correlation test, beta: r = −0.59, p = 0.03; alpha: r = −0.42, p = 0.16). **d** Comparison of M1-M1 PLV derived from actual and phase-shuffled surrogate data. The distribution of PLV was significantly altered after the permutation (Grand-average of surrogate data was shown). The null distribution

of PLV values indicates the original difference was significant in the permutation test (Beta: $p < 0.05$, Alpha: $p < 0.05$).

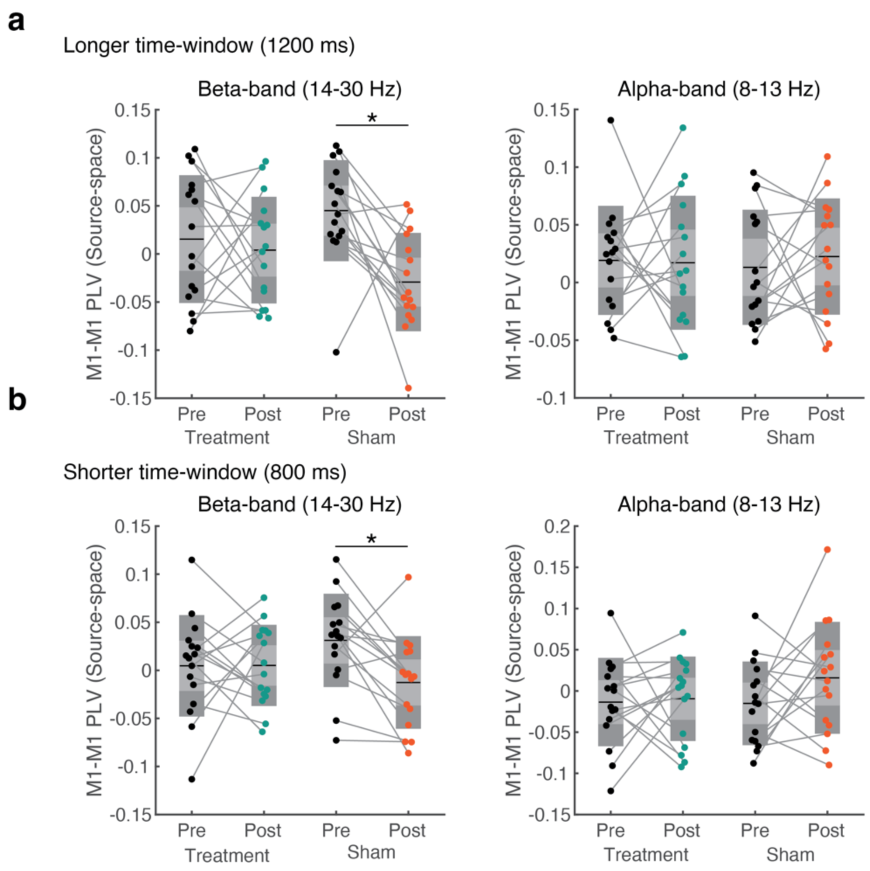

Supplementary Fig. 5 Supplementary analysis using alternative time-windows in Experiment 2

a Pre-movement M1-M1 PLVs in the beta-band calculated with a longer time-window (1200 ms). Differences in phase shuffled data were subjected to a mixed two-way repeated measures ANOVA. A significant interaction of "Time" × "Group" was observed in the beta-band ($F(1, 28) = 4.37$, $p = 0.045$, $\eta^2 = 0.074$) while no main effects of "Time" or "Group" were found ("Time": $F(1, 28) = 2.40$, $p = 0.13$, "Group": $F(1 , 28) = 0.019$, $p = 0.89$). Significant difference in the sham group revealed by post hoc *t*-tests (*:$p < 0.05$, $t(15) = 3.61$, $p = 0.003$, $d = 0.90$, CI_{95} = [0.31, 1.49]) For the identical analysis on the alpha-band data, no significant interaction and main effects were found (all $p > 0.05$). **b**

Pre-movement M1-M1 PLVs in the beta-band calculated with a shorter time-window (800 ms). Differences in phase shuffled data were subjected to a mixed two-way repeated measures ANOVA. A significant interaction of “Time” × ”Group” was observed in the beta-band ($F(1, 28) = 4.24$, $p = 0.049$, $\eta^2 = 0.06$) while no main effects of “Time” or “Group” were found (“Time”: $F(1, 28) = 0.11$, $p = 0.75$, “Group”: $F(1 , 28) = 0.11$, $p = 0.74$). Significant difference in the sham group revealed by post hoc *t*-tests (*:$p < 0.05$, $t(15) = 3.43$, $p = 0.004$, $d = 0.86$, CI_{95} = [0.27 1.42]) For the identical analysis on the alpha-band data, no significant interaction and main effects were found (all $p > 0.05$).

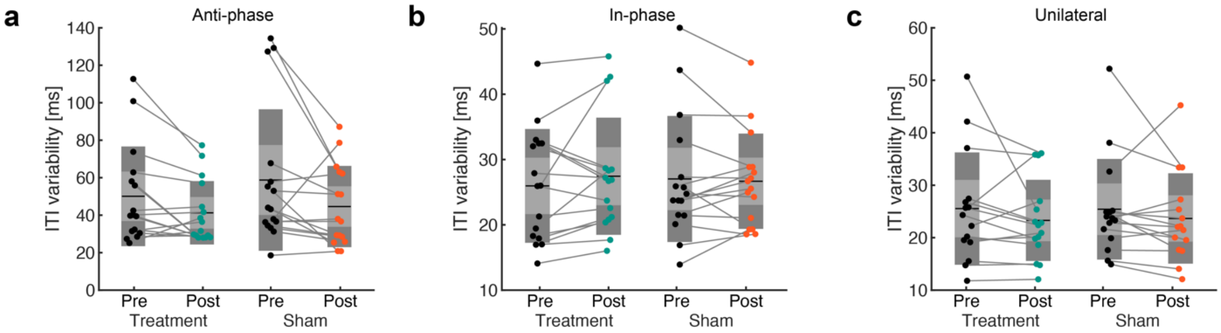

Supplementary Fig. 6 Changes in the variability of inter-tap intervals (ITI)

a ITI variability during the anti-phase bimanual tapping task. A two-way repeated measures ANOVA revealed significant main effect of “Time” ($F(1, 30) = 8.74$, $p = 0.006$, $\eta^2 = 0.23$), but not a main effect of “Group” ($F(1, 30) = 0.50$, $p = 0.49$) or their interaction ($F(1, 30) = 0.48$, $p = 0.50$). A post hoc *t*-test revealed significant decrease in the ITI variability ($t(30) = 2.98$, $p = 0.006$). **b** ITI variability during the in-phase bimanual tapping task. No significant main effects or interaction were observed (main effect of “Time”: $F(1, 30) = 0.41$, $p = 0.53$; “Group”: $F(1, 30) = 0.002$, $p = 0.96$; interaction of “Time” × “Group”: $F(1, 30) = 1.06$, $p = 0.31$). **c** ITI variability during unilateral tapping task. No significant main effects or interaction were observed (main effect of “Time”: $F(1, 28) = 2.40$, $p = 0.13$; “Group”: $F(1, 28) = 0.002$, $p = 0.97$; interaction of “Time” × “Group”: $F(1, 28) = 0.04$, $p = 0.85$). Two participants were excluded from the analysis due to data corruption.

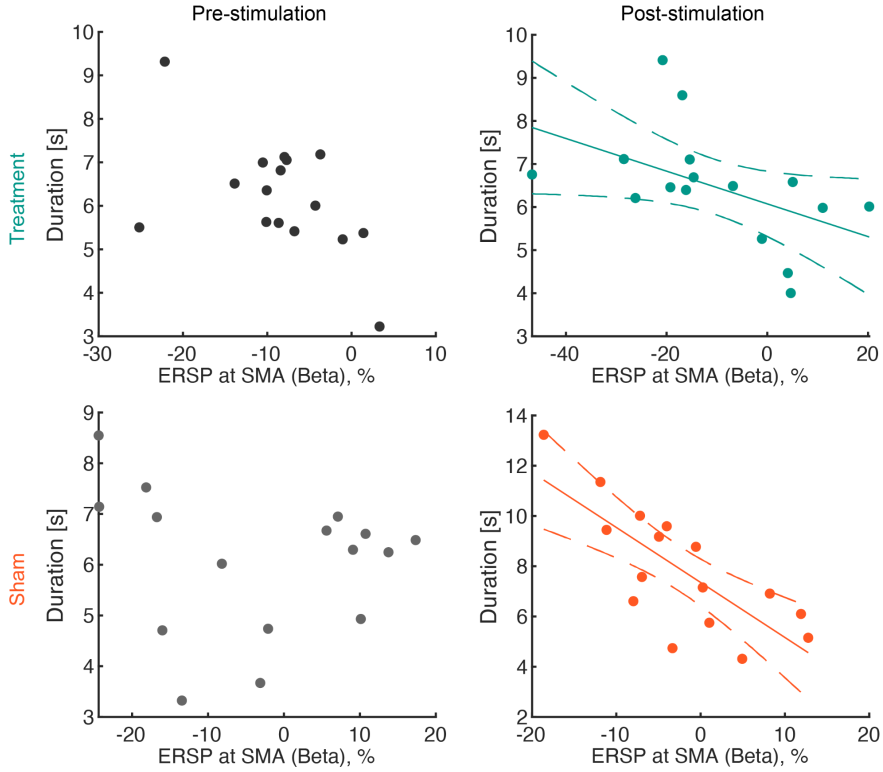

Supplementary Fig. 7 Cross-experiment replication of correlation between pre-movement beta-band activities in SMA and duration

Top Left: Pre-stimulation data from the treatment group (Spearman's correlation test, ρ = −0.418, p = 0.11, $CI_{95} = [-0.76, 0.10]$). *Top Right*: Post-stimulation data from the treatment group (r = −0.624, p = 0.012, $CI_{95} = [-0.85,\ -0.19]$). *Bottom Left*: Pre-stimulation data from the sham group (r = −0.297, p = 0.26, $CI_{95} = [-0.69, 0.23]$). *Bottom Right:* Post-stimulation data from the sham group (r = -0.756, p = 0.001, $CI_{95} = [-0.91, -0.42]$).

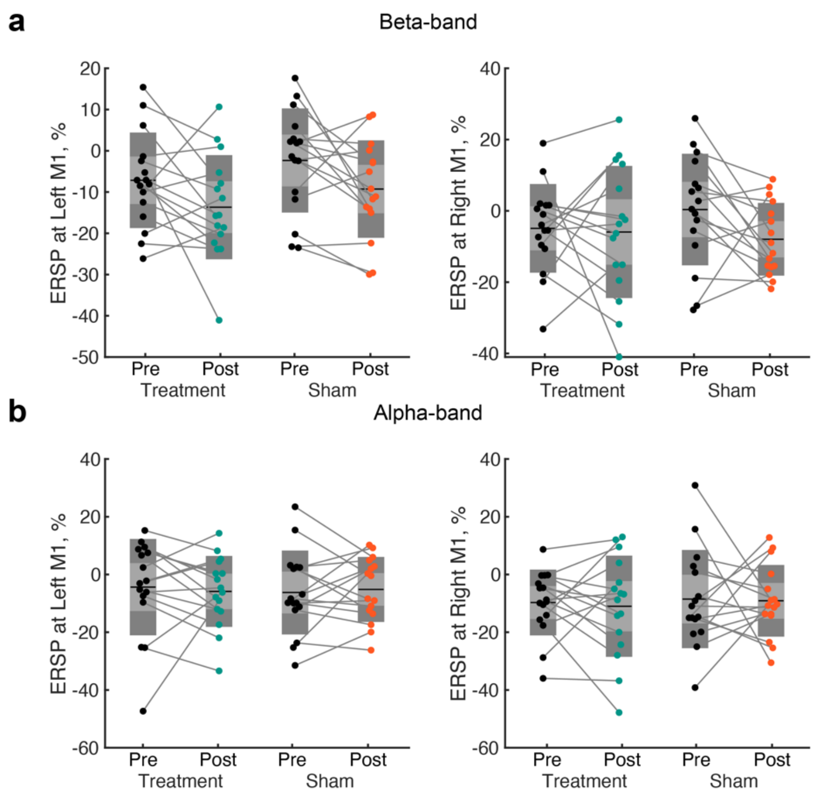

Supplementary Fig. 8 Changes in pre-movement ERSP magnitude at bilateral M1

a ERSP magnitude in the beta-band. *Left*: Data from left M1. A two-way repeated measures ANOVA revealed significant main effect of “Time” (*F(1,* 30) = 8.36, *p* = 0.006, η^2 = 0.07), but no main effect of “Group” (*F(1,* 30) = 1.67, *p* = 0.206) or their interaction (*F*(1, 30) = 0.008, *p* = 0.930). Post hoc *t*-test revealed significant decrease in ERSP magnitude (*t*(31) = 2.94, *p* = 0.006, *d* = 0.519, $CI_{95} = (0.15, -0.89)$). *Right*: Data from right M1. A two-way repeated measures ANOVA revealed no significant effect of “Time” (*F(1,* 30) = 1.97, *p* = 0.17), “Group” (*F(1,* 30) = 1.97, *p* = 0.171), or their interaction (*F(1,* 30) = 0.177, *p* = 0.68. **b** ERSP magnitude in the alpha-band. *Left*: Data from left M1. A two-way repeated measures ANOVA revealed no significant effects of “Time” (*F(1,* 30) = 0.007, *p* = 0.93), “Group” (*F(1,* 30) = 0.02, *p* = 0.90), or their interaction (*F(1,* 30) = 0.26, *p* = 0.61). *Right*: Data from right M1. A two-way repeated measures ANOVA revealed no significant effects of “Time” (*F(1,* 30) = 0.078, *p* = 0.78), “Group” (*F(1,* 30) = 0.157, *p* = 0.70), or their interaction (*F(1,* 30) = 0.011, *p* = 0.92).

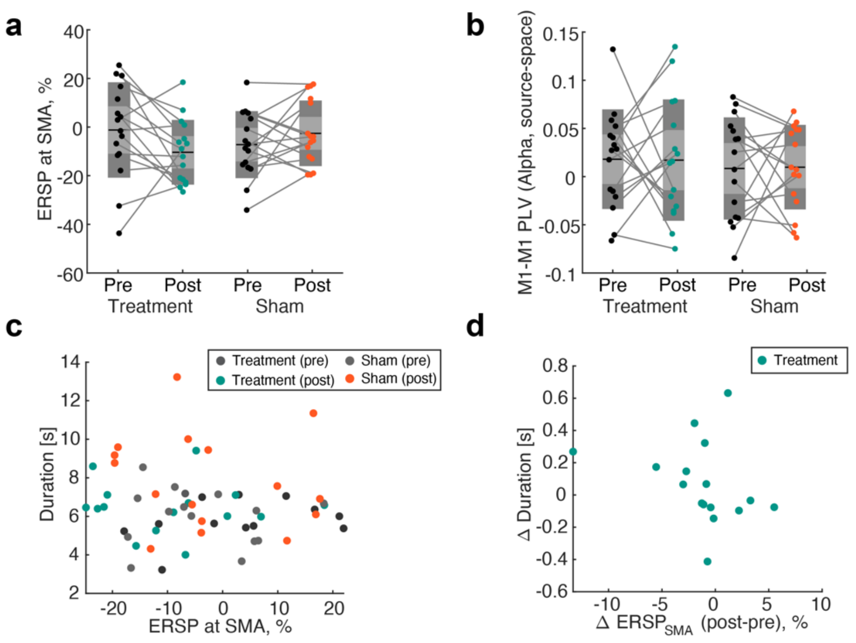

Supplementary Fig. 9 Pre-movement alpha activities at SMA and its behavioral association

a ERSP magnitude in the alpha-band. A two-way repeated measures ANOVA revealed significant interaction of “Time” × “Group” ($F(1, 30) = 4.71$, p = 0.038, $\eta^2 = 0.134$), but no main effect of “Time” ($F(1, 30) = 0.505$, $p = 0.48$) or “Group” ($F(1, 30) = 0.043$, $p = 0.84$). Post hoc t-test did not reveal any significant difference (treatment: $t(15) = -1.85$, $p = 0.084$; sham: $t(15) = 1.16$, $p = 0.26$; pre-stimulation: $t(30) = 1.02$, $p = 0.315$; post-stimulation: $t(30) = -1.681$, $p = 0.103$). **b** Pre-movement M1-M1 PLVs in the alpha-band. The difference from phase shuffled data were subjected to two-way repeated measures ANOVA. Significant effects were not found for interaction of “Time” × ”Group” ($F(1, 28) < 0.001$, $p = 0.99$) or main effect of “Time” ($F(1, 28) = 1.54$, $p = 0.23$) or “Group” ($F(1, 28) = 0.33$, $p = 0.57$). **c** Correlation analysis between pre-movement beta-band activities in SMA and duration. Pre-stimulation data from the treatment group (Pearson’s correlation test, $r = 0.259$, $p = 0.39$). Post-stimulation data from the treatment group ($r = -0.063$, $p = 0.83$). Pre-stimulation data from the sham group ($r = -0.101$, $p = 0.73$). Post-stimulation data from the sham group ($r = -0.174$, $p = 0.52$). **d** Correlation analysis of changes in duration

and SMA-ERSP magnitude in the alpha-band in the treatment group (Spearman's rank correlation test, $\rho = -0.468$, $p = 0.07$).

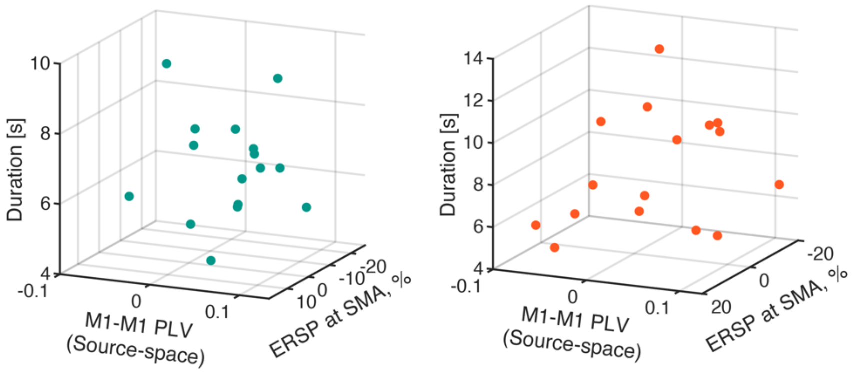

Supplementary Fig. 10. Linear mixed-effect model considering cortical activity patterns in alpha-band

Left: Data from the treatment group (ERSP × PLV: $p = 0.68$; ERSP: $p = 0.86$, M1-M1 PLV: $p = 0.97$). *Right:* Data from the sham group (ERSP × PLV: $p = 0.14$; ERSP: $p = 0.50$, M1-M1 PLV: $p = 0.90$).

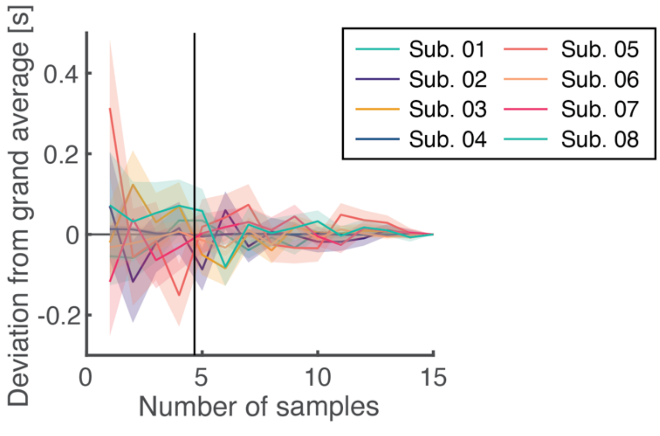

Supplementary Fig. 11: Variance of anti-phase duration for a dataset of preliminary experiments.

Each line indicates the average duration of data subsampled from the whole data (x-axis) for each participant (Shades indicate the standard error computed using the iteratively subsampled dataset), and the deviation from the grand average (second, y-axis), computed using the whole data. We set the threshold which exhibited 1.96 SE (the standard error of the difference from the grand average) as less than 2 s (10% of task duration). As indicated in the black line, 4.7 trials were necessary.

Supplementary Table 1. Studies incorporated in the automated meta-analysis by NeuroQuery

No	Paper information
1	Gavazzi, G. *et al.* Alexithymic trait is associated with right IFG and pre-SMA activation in non-emotional response inhibition in healthy subjects. *Neurosci. Lett.* **658**, 150–154 (2017).
2	Labriffe, M. *et al.* Brain activity during mental imagery of gait versus gait-like plantar stimulation: A novel combined functional MRI paradigm to better understand cerebral gait control. *Front. Hum. Neurosci.* **11**, (2017).
3	Meffert, H., Hwang, S., Nolan, Z. T., Chen, G. & Blair, J. R. Segregating attention from response control when performing a motor inhibition task. Segregating attention from response control. *Neuroimage* **126**, 27–38 (2016).
4	Leek, E. C., Yuen, K. S. L. & Johnston, S. J. Domain General Sequence Operations Contribute to Pre-SMA Involvement in Visuo-spatial Processing. *Front. Hum. Neurosci.* **10**, (2016).
5	Wu, T. *et al.* Attention to Automatic Movements in Parkinson's Disease: Modified Automatic Mode in the Striatum. *Cereb. Cortex* **25**, 3330–3342 (2015).
6	Censor, N., Dayan, E. & Cohen, L. G. Cortico-subcortical neuronal circuitry associated with reconsolidation of human procedural memories. *Cortex.* **58**, 281–288 (2014).
7	Hu, S., Tseng, Y. C., Winkler, A. D. & Li, C. S. R. Neural bases of individual variation in decision time. *Hum. Brain Mapp.* **35**, 2531–2542 (2014).
8	Wei, L. *et al.* Reduced topological efficiency in cortical-basal ganglia motor network of parkinson's disease: A resting state fMRI Study. *PLoS One* **9**, (2014).
9	Pecenka, N., Engel, A. & Keller, P. E. Neural correlates of auditory temporal predictions during sensorimotor synchronization. *Front. Hum. Neurosci.* **7**, (2013).
10	Yoshida, Y. *et al.* The neural substrates of the warning effect: a functional magnetic resonance imaging study. *Neurosci. Res.* **76**, 230–239 (2013).
11	Tremblay, P., Dick, A. S. & Small, S. L. Functional and structural aging of the speech sensorimotor neural system: functional magnetic resonance imaging evidence. *Neurobiol. Aging* **34**, (2013).
12	Schwartze, M., Rothermich, K. & Kotz, S. A. Functional dissociation of pre-SMA and SMA-proper in temporal processing. *Neuroimage* **60**, 290–298 (2012).
13	Lefebvre, S., Dricot, L., Gradkowski, W., Laloux, P. & Vandermeeren, Y. Brain activations underlying different patterns of performance improvement during early motor skill learning. *Neuroimage* **62**, 290–299 (2012).

14 Linortner, P. *et al.* White matter hyperintensities alter functional organization of the motor system. *Neurobiol. Aging* **33**, 197.e1-197.e9 (2012).

15 Keren, R. K. *et al.* Enhanced functional synchronization of medial and lateral PFC underlies internally-guided action planning. *Front. Hum. Neurosci.* **6**, 1–26 (2012).

16 Wu, T. *et al.* Effective connectivity of brain networks during self-initiated movement in Parkinson's disease. *Neuroimage* **55**, 204–215 (2011).

17 Wong, D., Dzemidzic, M., Talavage, T. M., Romito, L. M. & Byrd, K. E. Motor control of jaw movements: An fMRI study of parafunctional clench and grind behavior. *Brain Res.* **1383**, 206–217 (2011).

18 Coombes, S. A., Corcos, D. M. & Vaillancourt, D. E. Spatiotemporal tuning of brain activity and force performance. *Neuroimage* **54**, 2226–2236 (2011).

19 Yanaka, H. T., Saito, D. N., Uchiyama, Y. & Sadato, N. Neural substrates of phasic alertness: a functional magnetic resonance imaging study. *Neurosci. Res.* **68**, 51–58 (2010).

20 Brendel, B. *et al.* The contribution of mesiofrontal cortex to the preparation and execution of repetitive syllable productions: an fMRI study. *Neuroimage* **50**, 1219–1230 (2010).

21 Kraft, E. *et al.* Levodopa-induced striatal activation in Parkinson's disease: a functional MRI study. *Parkinsonism Relat. Disord.* **15**, 558–563 (2009).

22 Simmonds, D. J., Pekar, J. J. & Mostofsky, S. H. Meta-analysis of Go/No-go tasks demonstrating that fMRI activation associated with response inhibition is task-dependent. *Neuropsychologia* **46**, 224–232 (2008).

23 Inoue, M., Masaoka, Y., Kawamura, M., Okamoto, Y. & Homma, I. Differences in areas of human frontal medial wall activated by left and right motor execution: dipole-tracing analysis of grand-averaged potentials incorporated with MNI three-layer head model. *Neurosci. Lett.* **437**, 82–87 (2008).

24 Galléa, C., Graaf, J. B. de, Pailhous, J. & Bonnard, M. Error processing during online motor control depends on the response accuracy. *Behav. Brain Res.* **193**, 117–125 (2008).

25 Simmonds, D. J. *et al.* Functional brain correlates of response time variability in children. *Neuropsychologia* **45**, 2147–2157 (2007).

26 Christmann, C., Koeppe, C., Braus, D. F., Ruf, M. & Flor, H. A simultaneous EEG-fMRI study of painful electric stimulation. *Neuroimage* **34**, 1428–1437 (2007).

27 Tremblay, P. & Gracco, V. L. Contribution of the frontal lobe to externally and internally specified verbal responses: fMRI evidence. *Neuroimage* **33**, 947–957 (2006).

28 Alario, F. X., Chainay, H., Lehericy, S. & Cohen, L. The role of the supplementary motor area (SMA) in word production. *Brain Res.* **1076**, 129–143 (2006).

29 Zentgraf, K. *et al.* Differential activation of pre-SMA and SMA proper during action observation: effects of instructions. *Neuroimage* **26**, 662–672 (2005).

30 Henry, R. G., Berman, J. I., Nagarajan, S. S., Mukherjee, P. & Berger, M. S. Subcortical pathways serving cortical language sites: initial experience with diffusion tensor imaging fiber tracking combined with intraoperative language mapping. *Neuroimage* **21**, 616–622 (2004).
