# Supplementary material for: Beta rhythmicity in human motor cortex reflects neural population coupling that modulates subsequent finger coordination stability
Source: Commun Biol. 2022 Dec 15;5:1375. doi: 10.1038/s42003-022-04326-4 (PMC9755311; doi:10.1038/s42003-022-04326-4)
Supplement: Supplementary file 3 — Description of Additional Supplementary Files [file 42003_2022_4326_MOESM3_ESM.pdf]

Description of Additional Supplementary Files

File name: Supplementary Movie 1
Description: A typical example of spontaneous phase transition during antiphase coupling.
