# Supplementary material for: Beta rhythmicity in human motor cortex reflects neural population coupling that modulates subsequent finger coordination stability
Source: Commun Biol. 2022 Dec 15;5:1375. doi: 10.1038/s42003-022-04326-4 (PMC9755311; doi:10.1038/s42003-022-04326-4)
Supplement: Supplementary file 5 — Reporting Summary [file 42003_2022_4326_MOESM5_ESM.pdf]

nature portfolio

Corresponding author(s): Junichi Ushiba

Last updated by author(s): 2022/10/03

Reporting Summary

Nature Portfolio wishes to improve the reproducibility of the work that we publish. This form provides structure for consistency and transparency in reporting. For further information on Nature Portfolio policies, see our Editorial Policies and the Editorial Policy Checklist.

Statistics

For all statistical analyses, confirm that the following items are present in the figure legend, table legend, main text, or Methods section.

n/a	Confirmed	
☐	☒	The exact sample size (*n*) for each experimental group/condition, given as a discrete number and unit of measurement
☐	☒	A statement on whether measurements were taken from distinct samples or whether the same sample was measured repeatedly
☐	☒	The statistical test(s) used AND whether they are one- or two-sided<br>*Only common tests should be described solely by name; describe more complex techniques in the Methods section.*
☐	☒	A description of all covariates tested
☐	☒	A description of any assumptions or corrections, such as tests of normality and adjustment for multiple comparisons
☐	☒	A full description of the statistical parameters including central tendency (e.g. means) or other basic estimates (e.g. regression coefficient) AND variation (e.g. standard deviation) or associated estimates of uncertainty (e.g. confidence intervals)
☐	☒	For null hypothesis testing, the test statistic (e.g. *F*, *t*, *r*) with confidence intervals, effect sizes, degrees of freedom and *P* value noted<br>*Give P values as exact values whenever suitable.*
☒	☐	For Bayesian analysis, information on the choice of priors and Markov chain Monte Carlo settings
☒	☐	For hierarchical and complex designs, identification of the appropriate level for tests and full reporting of outcomes
☐	☒	Estimates of effect sizes (e.g. Cohen's *d*, Pearson's *r*), indicating how they were calculated

Our web collection on statistics for biologists contains articles on many of the points above.

Software and code

Policy information about availability of computer code

Data collection	MATLAB (Mathworks Inc.)
Data analysis	MATLAB (Mathworks Inc.), R, JASP

For manuscripts utilizing custom algorithms or software that are central to the research but not yet described in published literature, software must be made available to editors and reviewers. We strongly encourage code deposition in a community repository (e.g. GitHub). See the Nature Portfolio guidelines for submitting code & software for further information.

Data

Policy information about availability of data

All manuscripts must include a data availability statement. This statement should provide the following information, where applicable:

- Accession codes, unique identifiers, or web links for publicly available datasets
- A description of any restrictions on data availability
- For clinical datasets or third party data, please ensure that the statement adheres to our policy

All data are available in the main text, the supplementary materials or figshare (https://xxx)

Human research participants

Policy information about studies involving human research participants and Sex and Gender in Research.

Reporting on sex and gender	N/A
Population characteristics	N/A
Recruitment	Participants were recruited through an online questionnaire.
Ethics oversight	Ethics Committee of the Faculty of Science and Technology of Keio University (IRB approval number: 2021-133).

Note that full information on the approval of the study protocol must also be provided in the manuscript.

Field-specific reporting

Please select the one below that is the best fit for your research. If you are not sure, read the appropriate sections before making your selection.

☒ Life sciences ☐ Behavioural & social sciences ☐ Ecological, evolutionary & environmental sciences

For a reference copy of the document with all sections, see nature.com/documents/nr-reporting-summary-flat.pdf

Life sciences study design

All studies must disclose on these points even when the disclosure is negative.

Sample size	The sample size of experiment 1 was determined based on previous neuroimaging studies using a similar experimental paradigm aiming to test the within-participant effect of intervention9,13,15,29. Since that large effect size (at least Cohen's d = 1.0) was consistently reported, we calculated that 13 participants were needed based on a priori power analysis using G*Power 3.185 (α = 0.05, 1-β = 0.8 , Bonferroni corrected). Finally, we recruited 15 participants for each group by considering exclusion due to the insufficient number of trials for statistical analyses (assumed 20% dropout rate). That of experiment 2 was calculated from an a priori power analysis based on the effect size of changes in behavioral performance observed in experiment 1 (Cohen's d = 0.78, Fig. 1d); we calculated that 15 participants were needed for each group.
Data exclusions	No participants were excluded
Replication	We replicated the findings of Experiment 1 in Experiment 2.
Randomization	Participants were randomly allocated to experimental conditions.
Blinding	Participants were blinded to their experimental conditions.

Reporting for specific materials, systems and methods

We require information from authors about some types of materials, experimental systems and methods used in many studies. Here, indicate whether each material, system or method listed is relevant to your study. If you are not sure if a list item applies to your research, read the appropriate section before selecting a response.

Materials & experimental systems

n/a	Involved in the study	
☒	☐	Antibodies
☒	☐	Eukaryotic cell lines
☒	☐	Palaeontology and archaeology
☒	☐	Animals and other organisms
☒	☐	Clinical data
☒	☐	Dual use research of concern

Methods

n/a	Involved in the study	
☒	☐	ChIP-seq
☒	☐	Flow cytometry
☒	☐	MRI-based neuroimaging
